# Case Report: Re-irradiation of out-of-field recurrence of malignant phyllodes tumors of the breast after adjuvant radiotherapy

**DOI:** 10.3389/fonc.2026.1694960

**Published:** 2026-01-29

**Authors:** Ziwei Li, Juan Zhong, Yingying Shi, Wenliang Lu, Yanjv Lu, Ning Ge

**Affiliations:** 1 Department of Oncology, Maternal and Child Health Hospital of Hubei Province, Tongji Medical College, Huazhong University of Science and Technology, Wuhan, China; 2 Department of Thyroid and Breast Surgery, Maternal and Child Health Hospital of Hubei Province, Tongji Medical College, Huazhong University of Science and Technology, Wuhan, Hubei, China; 3 Department of Pathology, Maternal and Child Health Hospital of Hubei Province, Tongji Medical College, Huazhong University of Science and Technology, Wuhan, Hubei, China

**Keywords:** breast sarcoma, malignant phyllodes tumor, radiotherapy, regional recurrence, re-irradiation

## Abstract

**Introduction:**

Phyllodes tumor of the breast (PTB) is a rare fibroepithelial neoplasm, classified as benign, borderline, and malignant. They typically present as solitary, painless, firm masses with potential rapid enlargement, and 20% exceed 10 cm, often indicating malignancy. Local recurrence frequently occurs within two years postoperatively. While the survival benefit of adjuvant radiotherapy remains controversial, it significantly reduces recurrence rates. We report a 27×14 cm malignant PTB and evaluate optimal radiotherapy strategies and re-irradiation safety in recurrent cases.

**Case report:**

A 55-year-old female presented with a rapidly enlarging left breast mass, initially detected seven years prior, measuring 50×40 cm on physical examination. The patient underwent complete surgical excision, with a postoperative pathological mass measuring 27×14 cm, followed by adjuvant radiotherapy (50 Gy/25 fractions). One month after radiation, a 15×15 cm axillary recurrence was resected (10×8 cm specimen). Two months later, a 10×8 cm infraclavicular recurrence was excised (6×5 cm specimen). Subsequently, six cycles of epirubicin–cyclophosphamide chemotherapy and re-irradiation (45–60 Gy/25 fractions) were administered. At present, there is no evidence of local recurrence.

**Conclusions:**

This case highlights the potential role of adjuvant RT in reducing recurrence and the feasibility of carefully selected re-irradiation for recurrent MPTs. Prospective studies are needed to define optimal target volume of radiotherapy, dose fractionation, and the safety of re-irradiation.

## 1 Introduction

Phyllodes tumor of the breast (PTB) is a rare type of breast tumor, accounting for 0.3%–1.0% of all breast neoplasms (1). Clinically, it predominantly occurs in women aged 35–55 years, with Asian women exhibiting a notably earlier age of onset compared to women of other ethnicities (2). PTB is classified into three types: benign PTB (accounting for approximately 60%), borderline PTB (accounting for approximately 15%), and malignant PTB (accounting for approximately 25%) (3, 4). PTB typically presents as a solitary, painless, firm mass in one breast, without skin adhesion, and may exhibit progressive growth. The incidence is roughly equal between the left and right breasts, with approximately 1% of cases involving bilateral involvement, either synchronously or metachronously. The onset is often insidious, and the tumor size may remain stable for years but can also undergo rapid enlargement within months. Approximately 20% of PTBs exceed 10 cm in diameter, with larger tumors frequently suggesting malignant PTB (5, 6). Recurrence of PTB most frequently occurs within two years postoperatively, predominantly at the surgical site. A large-scale analysis of 3,120 patients (7) demonstrated that while adjuvant radiotherapy did not improve disease-free survival (DFS) or overall survival (OS), it significantly reduced the local recurrence rate. In this study, we present a rare case of a giant malignant phyllodes tumor measuring 27×14 cm and discuss the optimal radiotherapy target volume for malignant phyllodes tumors of the breast, and to evaluate the safety and efficacy of re-irradiation in cases of multiple local recurrences.

## 2 Case report

**Figure 1:**
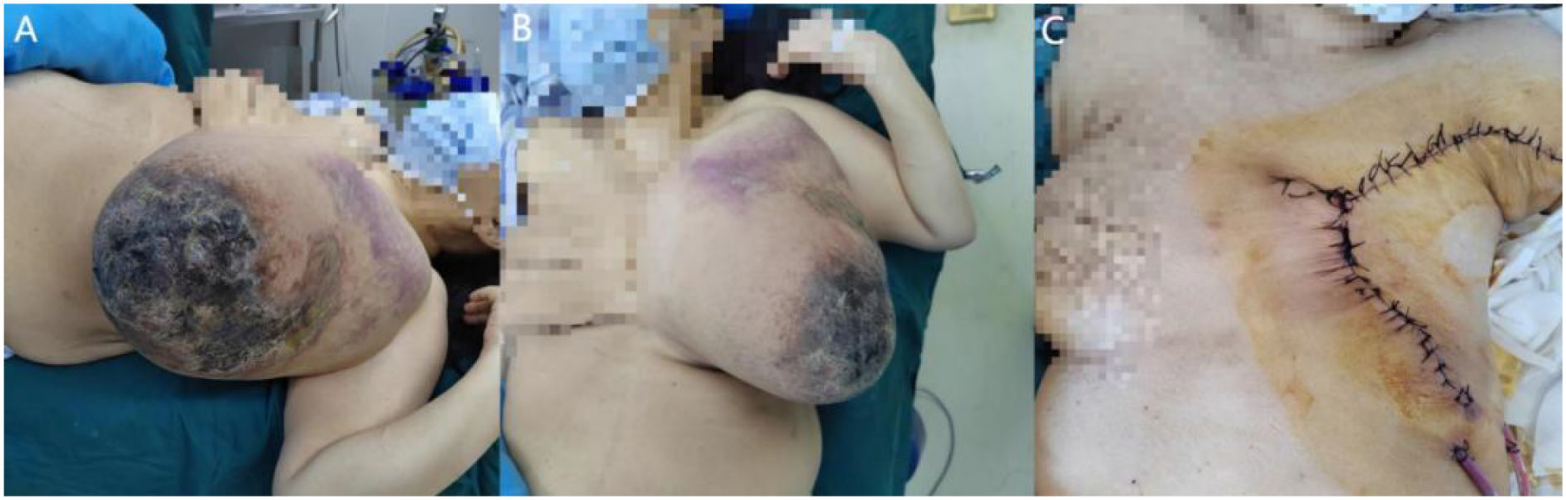
Intraoperative findings of the large malignant phyllodes tumor. **(A, B)** Preoperative tumor appearance; **(C)** surgical site following excision and primary closure.

**Figure 2:**
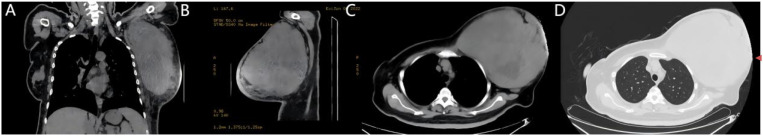
Preoperative CT images of the left breast showing a giant heterogeneous mass. **(A–D)** correspond to coronal, sagittal, axial, and lung window views.

A 55-year-old woman who presented in June 2022, with a mass in the left breast discovered over seven years ago and suddenly increased in size one month later. On physical examination, a large palpable mass, approximately 50 cm × 40 cm, was detected in the left breast, accompanied by localized skin crusting, subcutaneous ecchymosis, and tenderness (**Figures 1A, B**). Multiple nodules were palpable in both breasts, particularly prominent in the upper outer quadrants. No enlarged superficial lymph nodes were detected in either axilla. Over 1 month, the mass progressively enlarged, which seriously affected the patient’s daily life. The patient had no family history of breast tumors. Preoperative CT imaging was performed (**Figure 2**).

**Figure 3:**
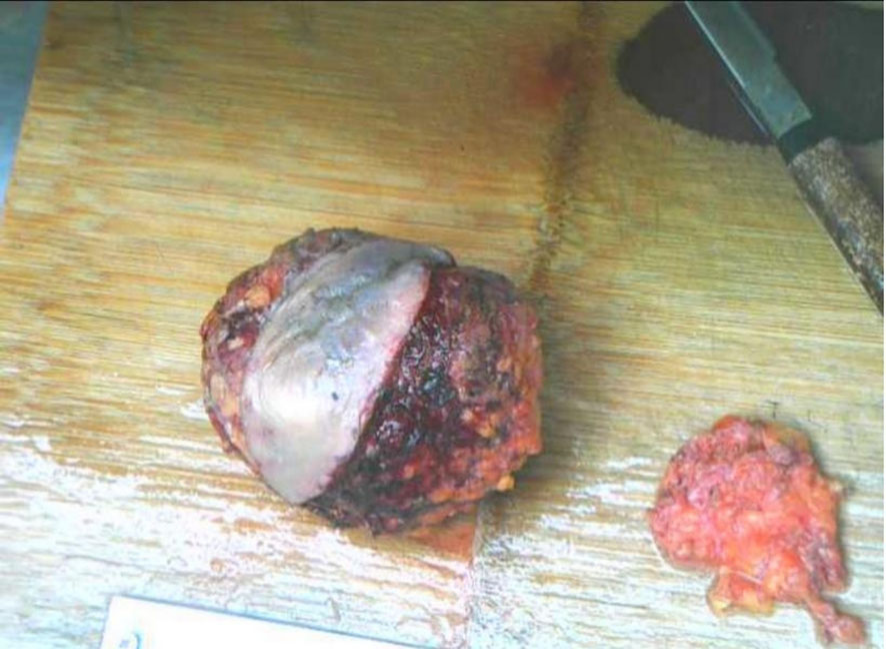
Macroscopic surgical specimen.

A transverse elliptical incision (approximately 20 cm in length) including the nipple-areolar complex was made on the left breast. The tumor occupied the entire breast parenchyma (approximately 50 × 40 cm) with ill-defined borders. Resection was performed with a minimum 3 cm macroscopic margin. Skin and subcutaneous tissue were incised, and thin flaps (approximately 0.3-0.4 cm in thickness) were elevated. Dissection extended superiorly to the clavicle, medially to the parasternal line, laterally to the anterior edge of the latissimus dorsi, and inferiorly to the costal margin. After incising the superficial pectoral fascia, the entire breast parenchyma with pectoralis major fascia was sharply dissected from the muscle surface. The tumor was completely resected, with no obvious residual disease observed. Gross appearance of the tumor after resection(**Figure 3**). A left axillary lymph node dissection (levels I, II) was also performed concurrently. A total of five lymph nodes were resected. The skin was closed with a continuous subcuticular suture, and the specimen with regional lymph nodes were sent for histopathology (**Figure 1C**).

**Figure 4:**
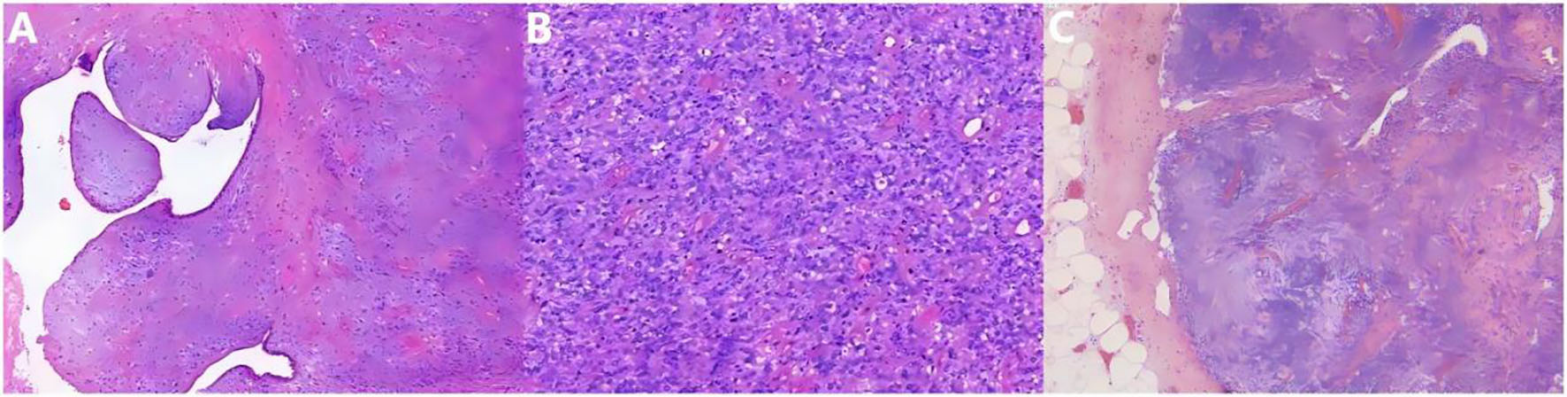
Hematoxylin and eosin-stained sections demonstrating the main morphological findings: **(A)** At low magnification (10×), proliferative stromal cells are observed, forming leaf-like projections. **(B)** The stromal cells exhibit moderate to marked atypical proliferation, with mitotic figures exceeding 10 per 10 high-power fields (HPF), and focal tumor necrosis is observed. **(C)** Pathologic examination of the submitted lymph nodes showed no tumor cells.

Malignant phyllodes tumor of the left breast with hemorrhagic infarction and degenerative changes. Key Features: Tumor characteristics: Size: 27cm×14 cm. Stromal atypia: Moderate to severe. Mitotic activity: >10 mitoses/10 HPF. No heterologous differentiation or definitive vascular tumor emboli identified. Invasion and secondary changes: Infiltration into subcutaneous breast tissue. Overlying ulceration and inflammatory changes in the epidermis. Margin status: Negative surgical margins (no tumor at inked resection borders). Five axillary lymph nodes were submitted for pathological examination, all of which were negative, as shown in **Figure 4C**. Immunohistochemistry(IHC): Epithelial markers: PCK(−), CK7(−), CK5/6(−). Myoepithelial markers: P63(−), Calponin(−). Hormone receptors: ER(−), PR(−). Others: HER2 (0), EGFR(+), Ki67 (LI~60%) (**Figure 4**). Based on the preoperative imaging studies and postoperative pathological findings, the clinical stage of this patient was determined to be T4N0M0, stage IIIB.

**Figure 5:**
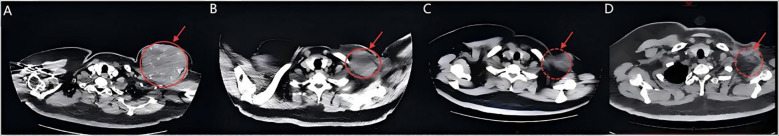
Clinical course of recurrence and re-irradiation. **(A)** Axillary recurrence at 6 weeks after initial RT. **(B)** Infraclavicular recurrence 2 months post-second resection. **(C)** Prior to re-irradiation. **(D)** No recurrence 3 months after re-irradiation.

In August 2022, the patient began adjuvant chest wall RT (50 Gy/25 fractions). Six weeks later, a 15 × 15 cm recurrent mass was detected in the left axilla. Surgical resection was performed in November 2022 (**Figure 5A**), yielding a 10 × 8 cm specimen. Three months later (February 2023), a second recurrence was detected in the left infraclavicular region (**Figure 5B**), and a 10 × 8 cm mass was excised. Pathology again confirmed malignant phyllodes tumor. Following the second recurrence, the patient received six cycles of epirubicin–cyclophosphamide chemotherapy. Two months after completion, in August 2023, re-irradiation was delivered to partial of the chest wall, infraclavicular and supraclavicular regions and axilla (**Figure 5C**). Prescribed doses were 45 Gy to a partial of the chest wall, axilla, infraclavicular and supraclavicular regions, and 60 Gy to suspicious nodes, all in 25 fractions. CT three months later showed no residual or recurrent disease (**Figure 5D**). At the latest follow-up, the patient remains free of local recurrence and systemic metastasis. She tolerated both courses of RT without severe late toxicity.

The re-irradiation plan utilized VMAT technique with 6 MV X-rays and the patient positioned supine and immobilized using a customized device.

Although the initial radiotherapy (RT) field encompassed only the chest wall, the axillary recurrence extended superiorly and laterally toward the lateral chest wall. Consequently, a partial of the chest wall—specifically the area adjacent to the recurrent axillary tumor bed was included in the re-irradiation volume to ensure adequate coverage of the involved margin.In addition, the recurrent lesion lay superior to the cranial border of the initial radiation field, further justifying the need to retreat this region. For the axillary recurrence, the target volume was delineated according to the precise location of the recurrent tumor bed as identified on imaging. The infraclavicular and supraclavicular regions were not included in the initial treatment. Given their function as key lymphatic drainage pathways for the axilla, prophylactic irradiation of these nodal basins was incorporated into the re-irradiation plan to reduce the risk of subsequent regional recurrence.

**Figure 6:**
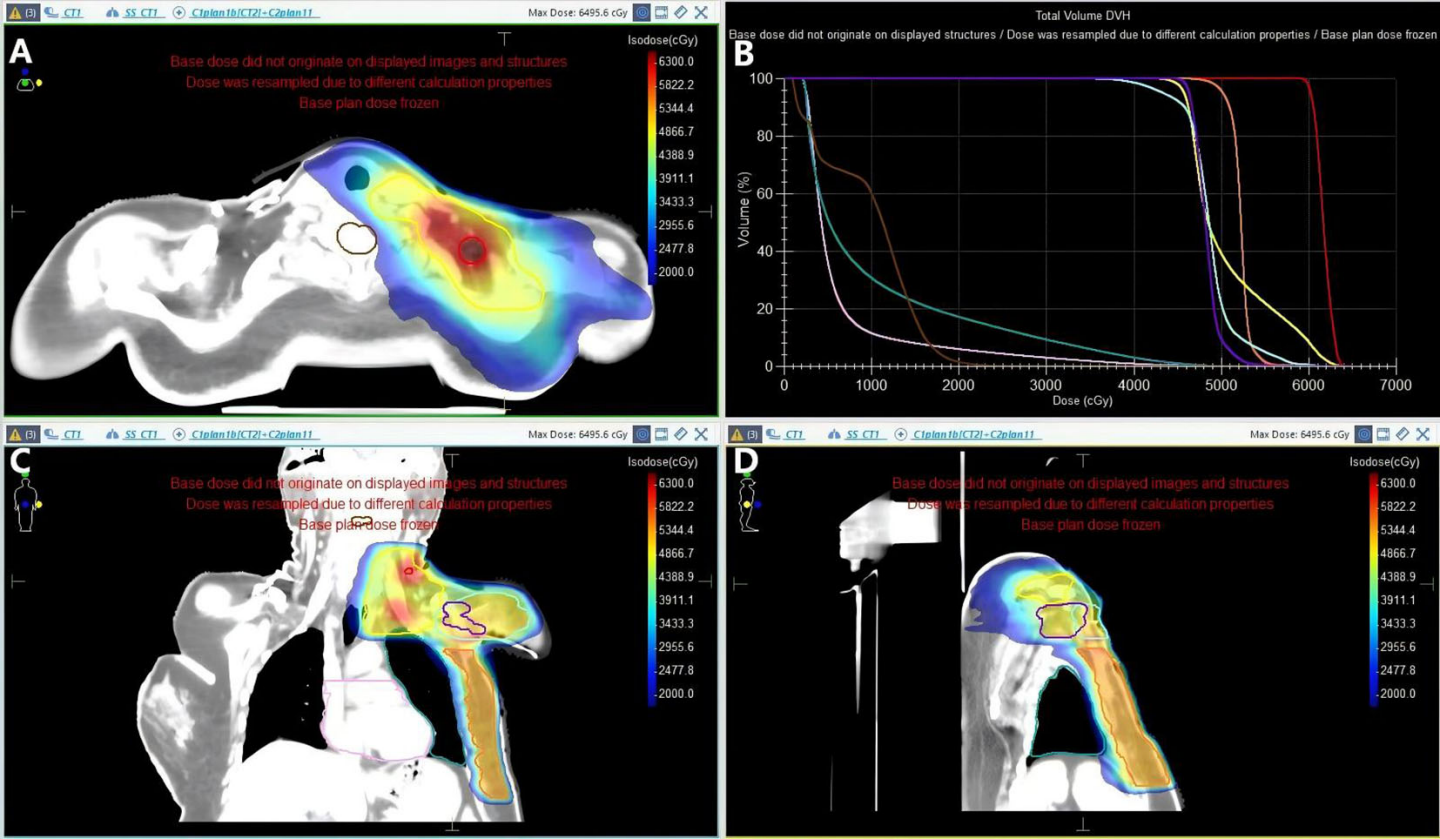
A composite dose map that overlays the initial RT plan and the re-irradiation plan. **(A)** Axial view; **(B)** Dose-Volume Histogram (DVH); **(C)** Coronal view; **(D)** Sagittal view:The orange contour represents the initial chest wall PTV. The yellow contour represents the re-irradiation PTV of the supraclavicular and infraclavicular regions; The light blue contour represents the re-irradiation PTV of the partial chest wall; The purple contour represents the re-irradiation PTV of the axilla; The red contour represents the PTV of the suspicious lymph node; The cyan contour represents the left lung; the pink contour represents the heart; and the brown contour represents the spinal cord.

For a partial of the chest wall irradiation, a 0.5-cm compensator was applied. The CT images from the initial radiotherapy plan were fused with those from the re-irradiation plan for target volume delineation (**Figure 6**).

Prescribed doses and field boundaries included:

Partial of the chest wall: Prescribed doses were 45 Gy in 25 fractions. The medial border is the midline, and the lateral border is the axillary midline. The superior border is at the level of the clavicular head, and the inferior border corresponding to the superior edge of the 10 Gy isodose line from the prior radiotherapy course. This objective isodose landmark was chosen to limit cumulative high-dose overlap, facilitate composite dose summation, and provide a conservative buffer for setup uncertainties.

Infraclavicular and supraclavicular region: Prescribed doses were 45 Gy in 25 fractions. The superior border is at the cricoid cartilage, the inferior border is at the inferior clavicle edge, with the medial border 1 cm medial to the midline and the sternocleidomastoid muscle, and the lateral border at the medial border of the humerus head.

Axillary region irradiation: Prescribed doses were 45 Gy in 25 fractions. Encompasses the entire axillary region.

Suspicious lymph nodes: Prescribed doses were 60 Gy in 25 fractions. Full coverage of at-risk regions.

Considering setup uncertainties, the first and second radiation fields overlapped by only approximately 1 cm (**Figure 6**), and the interval between the two treatments was one year, with all normal tissue doses remaining within acceptable tolerance limits. A standardized workflow for re-irradiation evaluation, the ReRT-SMPC (Retreatment Special Medical Physics Consult), was developed by Paradis et al. at the University of Michigan (8). Following this framework, prior doses to critical normal tissues were conservatively reduced: 50% for the heart and spinal cord, and 25% for the lungs, when estimating retreatment tolerance. After incorporating the planned re-irradiation dose, all composite OAR doses remained within safe thresholds.

Subsequent follow-up visits and imaging assessments have been conducted every three months during the two years after re-irradiation, and no recurrence has been observed to date.

## 3 Discussion

Malignant phyllodes tumors (MPTs) of the breast are rare fibroepithelial neoplasms with a propensity for local recurrence and, less commonly, distant metastasis. Surgical resection remains the cornerstone of treatment, with breast-conserving surgery (BCS) or mastectomy chosen based on tumor size and margin status (9). Wide local excision (≥1 cm margins) is recommended, yet recurrence rates remain high, especially in borderline and malignant subtypes. Local recurrence rates of 21–36% have been reported after margin-negative BCS for malignant tumors, compared to 8% for benign tumors. Mastectomy is often preferred for large tumors (>5 cm) or when negative margins are difficult to achieve (10). Despite aggressive surgery, recurrence remains a major concern, necessitating consideration of adjuvant therapies.

Although the surgical margins were negative, the possibility of microscopic residual disease cannot be completely excluded. This may partly explain the rapid postoperative enlargement and the subsequent out-of-field recurrence observed in this case. The markedly elevated Ki-67 index (11), together with a mitotic count exceeding 10 mitoses per 10 high-power fields, indicates a highly aggressive tumor phenotype. Increased Ki-67 expression is strongly associated with a higher risk of local recurrence and poorer prognosis. This high-risk profile further supports the clinical value of adjuvant radiotherapy.

The role of adjuvant radiotherapy (RT) in improving outcomes remains debated (12–14). Several retrospective studies and meta-analyses support its efficacy in reducing local recurrence. A systematic review of 2,708 patients reported significant benefit (HR = 0.43, 95% CI: 0.23–0.64), particularly following BCS (HR = 0.31) (15). Prospective data by Barth et al. showed no local recurrences in 46 patients treated with BCS plus whole-breast RT at a median 56-month follow-up (10). Another series of 63 cases found that combined surgery and RT yielded survival of 60–70 months, even in patients with multiple recurrences (16). These findings suggest RT may compensate for limited surgical margins, though the lack of survival benefit raises questions about whether it delays rather than prevents recurrence.

Optimal RT regimens remain undefined. Conventional fractionation (50–50.4 Gy in 25–28 fractions, with boosts to 60 Gy) is commonly used (17). Given that recurrences usually occur at the primary site, partial breast irradiation (PBI) has been explored. An ongoing prospective trial is evaluating PBI in borderline and malignant tumors, hypothesizing equivalent control with reduced toxicity (10). This underscores the urgent need for prospective, tumor-specific protocols.

Target volume delineation is another challenge. Some adopt a tumor-bed boost plus ~2 cm CTV expansion to account for infiltrative growth (12). A UK–Ireland survey revealed wide heterogeneity, ranging from whole-breast RT to partial breast or margin-directed RT (18). Others argue whole-breast RT may be unnecessary due to the rarity of multicentric disease (10). Further studies are needed to refine RT volumes and dosing strategies, especially in high-risk subgroups.

For recurrence, ESMO 2012 guidelines recommend consolidation RT in patients without prior RT, and re-irradiation in selected cases with prior RT, considering toxicity and recurrence risk (19). A retrospective study of 144 patients with first isolated locoregional recurrence found that surgery plus RT significantly reduced further recurrence, with chest wall and supraclavicular regions the most common relapse sites (20). NCCN guidelines similarly recommend RT for recurrent borderline or malignant tumors (21), with regimens of 50–60 Gy plus tumor bed boost commonly employed (22). Collectively, these data highlight the importance of tailoring re-irradiation based on recurrence pattern, prior dose, and patient factors to maximize control while limiting toxicity.

Although RT reduces local recurrence, its effect on survival remains uncertain. A SEER study found no OS or BCSS benefit after matching (14), suggesting that distant metastasis may play a greater role in determining mortality. Selecting appropriate candidates for RT is also challenging; younger patients and those with larger tumors may benefit more, but reliable biomarkers are lacking (7, 12). Emerging tools such as genomic profiling and ctDNA monitoring may eventually refine risk stratification, although these were not performed in our patient due to resource limitations.

In conclusion, while surgery remains the mainstay for MPTs, high recurrence rates necessitate ongoing investigation of adjuvant therapies. Prospective studies are needed to clarify the role of RT and identify patients most likely to benefit. Although direct evidence for re-irradiation is limited, cross-tumor guidelines and recurrent breast cancer data support its feasibility. ESMO consensus allows re-irradiation in selected recurrent cases (19), and breast cancer studies confirm acceptable toxicity and meaningful local control (23, 24). In the present case, second-course RT (45–60 Gy/25 fractions) following prior full-dose chest wall irradiation achieved durable local control, highlighting the exploratory potential of re-irradiation in recurrent MPTs.

## 4 Patient’s perspective

The patient provided fully informed consent for the publication of this report and the accompanying images. She reported being highly satisfied with the oncological outcome, functional recovery, and cosmetic results. She appreciated the detailed explanations and support provided by the multidisciplinary team throughout treatment and follow-up. The patient expressed relief and confidence in returning to daily activities, emphasizing the positive impact of attentive care on her overall well-being.

## Data Availability

The original contributions presented in the study are included in the article/supplementary material. Further inquiries can be directed to the corresponding author.
